# The Non-Invasive Diagnosis of Chronic Coronary Syndrome: A Focus on Stress Computed Tomography Perfusion and Stress Cardiac Magnetic Resonance

**DOI:** 10.3390/jcm12113793

**Published:** 2023-05-31

**Authors:** Léon Groenhoff, Giulia De Zan, Pietro Costantini, Agnese Siani, Eleonora Ostillio, Serena Carriero, Giuseppe Muscogiuri, Luca Bergamaschi, Giuseppe Patti, Carmine Pizzi, Sandro Sironi, Anna Giulia Pavon, Alessandro Carriero, Marco Guglielmo

**Affiliations:** 1 Radiology Department, Maggiore della Carità Hospital, 28100 Novara, Italy; leongroenhoff@gmail.com (L.G.); pietro.costantini.md@gmail.com (P.C.); agnesesiani@gmail.com (A.S.); eleonora.dott@gmail.com (E.O.); alessandro.carriero@maggioreosp.novara.it (A.C.); 2 Department of Translational Medicine, University of Eastern Piedmont, Maggiore della Carità Hospital, 28100 Novara, Italy; g.dezan@umcutrecht.nl (G.D.Z.); giuseppe.patti@maggioreosp.novara.it (G.P.); 3 Department of Cardiology, Division of Heart and Lungs, Utrecht University Medical Center, 3584 CX Utrecht, The Netherlands; 4 Postgraduate School in Radiodiagnostics, University of Milan, 20122 Milan, Italy; serena.carriero@unimi.it; 5 Department of Radiology, IRCCS Istituto Auxologico Italiano, San Luca Hospital, 20149 Milan, Italy; g.muscogiuri@gmail.com; 6 School of Medicine, University of Milano-Bicocca, 20900 Monza, Italy; sandro.sironi@unimib.it; 7 Cardiology Unit, Cardiac Thoracic and Vascular Department, IRCCS Azienda Ospedaliera-Universitaria di Bologna, 40138 Bologna, Italy; lucabergamaschi91@gmail.com (L.B.); carmine.pizzi@unibo.it (C.P.); 8 Department of Medical and Surgical Sciences—DIMEC, Alma Mater Studiorum, University of Bologna, 40126 Bologna, Italy; 9 Department of Radiology, ASST Papa Giovanni XXIII, 24127 Bergamo, Italy; 10 Cardiovascular Department, Cardiocentro Ticino Institute, Ente Ospedaliero Cantonale, 6900 Lugano, Switzerland; annagiulia.pavon@eoc.ch; 11 Department of Cardiology, Haga Teaching Hospital, 2545 AA The Hague, The Netherlands

**Keywords:** coronary artery disease, cardiac coronary syndrome, stress imaging, cardiac computed tomography perfusion, stress magnetic cardiac resonance

## Abstract

Coronary artery disease is still a major cause of death and morbidity worldwide. In the setting of chronic coronary disease, demonstration of inducible ischemia is mandatory to address treatment. Consequently, scientific and technological efforts were made in response to the request for non-invasive diagnostic tools with better sensitivity and specificity. To date, clinicians have at their disposal a wide range of stress-imaging techniques. Among others, stress cardiac magnetic resonance (S-CMR) and computed tomography perfusion (CTP) techniques both demonstrated their diagnostic efficacy and prognostic value in clinical trials when compared to other non-invasive ischemia-assessing techniques and invasive fractional flow reserve measurement techniques. Standardized protocols for both S-CMR and CTP usually imply the administration of vasodilator agents to induce hyperemia and contrast agents to depict perfusion defects. However, both methods have their own limitations, meaning that optimizing their performance still requires a patient-tailored approach. This review focuses on the characteristics, drawbacks, and future perspectives of these two techniques.

## 1. Introduction

Coronary artery disease (CAD) represents a major cause of mortality and morbidity [1]; nowadays, it accounts for a considerable proportion of healthcare costs, which are expected to double by 2030 [2]. In 2019, the European Society of Cardiology (ESC) published guidelines on the diagnosis and management of chronic coronary syndrome (CCS), recommending diagnostic strategies that are predominantly based on cardiac imaging [3]. Indeed, non-invasive imaging methods provide detection of the disease and can guide therapy and predict outcomes [4].

However, while coronary computed tomographic angiography (CCTA) has a known high-negative predictive value in ruling out CAD, at the same time, it has limited accuracy in the diagnosis of hemodynamically significant coronary artery stenosis, especially those graded between 30% and 70%, as anatomical data alone are not predictive of inducible ischemia [5]. Therefore, an additional functional test is often required because both anatomic and functional information is needed to guide patient care and revascularization. Cardiac stress imaging techniques include, among others, nuclear perfusion, stress echocardiography, stress myocardial computed tomography perfusion (CTP), and stress cardiovascular magnetic resonance (S-CMR). These non-invasive functional imaging modalities can help in assessing significant myocardial ischemia, myocardium viability, and exercise capacity. The initial diagnostic workup should consider the performance and availability of each of the different imaging tests, as well as the patient profile. Table 1 gives an overview of the main characteristics of the ischemia-assessing techniques at our disposal in terms of availability, cost, spatial and temporal resolution, sensitivity, and specificity.

Stress echocardiography remains the most available non-invasive imaging technique, which can detect ischemia without the use of ionizing radiation or contrast agents. However, its diagnostic accuracy is limited compared to more advanced imaging techniques.

S-CMR is a non-ionizing technique that can evaluate perfusion defects or ischemic wall motion abnormalities caused by pharmacological stress or even exercise. As will be explored later in more detail, several studies showed the high negative predictive value and high diagnostic accuracy of S-CMR to detect CAD compared to the gold standard techniques: angiographically determined luminal coronary stenosis and fractional flow reserve (FFR) [4]. The advantages of S-CMR are the lack of ionizing radiation and the relatively low cost. Nevertheless, S-CMR has disadvantages, such as its low availability and the high level of expertise required. 

CTP is a non-invasive imaging technique that requires the administration of a stress agent, followed by a CT acquisition, and evaluates myocardial ischemia over the coronary anatomy and plaque stenosis. Past studies demonstrated that using stress CTP increases the diagnostic accuracy of CCTA [6]. 

This literature review aims to describe the technical prerequisites, challenges, performance, protocols, and applications related to CTP and S-CMR. Moreover, we will discuss and define both of these techniques’ current state-of-the-art and future directions.

## 2. Stress Cardiac Magnetic Resonance

### 2.1. Why S-CMR

S-CMR is a functional non-invasive imaging test that has strongly consolidated its position in recent years, being able not only to highlight myocardial viability and cardiac function, but also to assess myocardial ischemia without the need for ionizing radiation [7]. 

Data from the multinational multicenter European Cardiovascular Magnetic Resonance registry demonstrated the safety of the procedure after analyzing the complications that occurred in 10,228 patients who underwent S-CMR. The results show that severe complications are extremely rare (0.07%), with only one patient suffering from anaphylactic shock due to adenosine. Mild complications were observed in 7.3% of the cases and manifestations included dyspnea, chest discomfort, ectopic beats attributed to the pharmacologic agent, and mild allergic reactions to Gadolinium-based contrast agents [8].

Studies evaluating the accuracy in diagnosing CAD in patients with suspected CCS date back to the MR Impact, which, in 2008, outlined the non-inferiority of S-CMR in comparison to single-photon emission computed tomography (SPECT) in detecting CAD, using invasive coronary angiography (ICA) as a gold standard and defining CAD as diameter stenosis > or =50% [9]. Subsequently, MR-Impact II also demonstrated S-CMR to have better sensitivity in diagnosing CAD compared to SPECT [10]. Moreover, the CE MARC study not only confirmed the higher sensitivity and negative predictive value of S-CMR compared to SPECT, but also showed similar specificity and positive predictive values of the two methods for detecting significant CAD, which was defined as 70% or more stenosis of a first order coronary artery (or left main stem stenosis of at least 50%) as measured via quantitative ICA [11]. The following CE MARC 2 trial in patients with suspected angina resulted in a lower probability of unnecessary ICA using S-CMR than NICE guideline-directed care [12]. Eventually, Arai et al. managed to demonstrate a higher specificity of S-CMR than SPECT in detecting single- and multi-vessel CAD (87% and 73%, respectively) [13]. These results from the GadaCAD supported the U.S. Food and Drug Administration’s approval of gadolinium-based contrast agents to assess myocardial perfusion and late gadolinium enhancement (LGE) in adult patients with known or suspected CAD. 

While all the above-mentioned studies used anatomic criteria to define the presence of CAD on ICA, multiple studies also demonstrated a high correlation between stress CMR perfusion interpreted qualitatively and invasive FFR [7]. When compared to other imaging modalities, S-CMR appears to have the highest diagnostic performance against FFR as the reference standard, both in terms of per-patient (sensitivity 90%, specificity 94%) and per-vessel (sensitivity 91%, specificity 85%) analyses [14]. Similarly, Pontone et al. published a meta-analysis comparing the diagnostic performance of multiple non-invasive imaging tests using invasive FFR as a reference standard and showed that, together with the combination of CCTA plus stress CT, S-CMR have the highest specificity in both vessel- and a patient-based models. Nevertheless, S-CMR demonstrated better performance in identifying patients who needed subsequent invasive coronary artery procedures [15]. 

The diagnostic efficiency of S-CMR is believed to be a result of better contrast, as well as spatial and temporal resolution. This efficiency permits us to assess perfusion within the different myocardium layers and, consequently, to identify even subendocardial defects, in contrast to SPECT, where the poorer spatial resolution implies that ischemic segments are best identified in the context of a normally perfused myocardial segment. Moreover, CMR is not limited by attenuation artifacts or contamination of the myocardium by signal sources not related to myocardial perfusion that can mimic or disguise perfusion defects. 

Recently, several studies also investigated the prognostic value of S-CMR. Among others, the retrospective multicenter SPINS trial demonstrated a >4-fold higher annual primary outcome of cardiovascular death or non-fatal myocardial infarction and a >10-fold higher rate of coronary revascularization during the first year after a positive S-CMR in comparison to patients without ischemia or late gadolinium enhancement (LGE) on CMR [16]. Similarly, Pezel et al. followed the occurrence of cardiovascular death or non-fatal myocardial infarction in patients with known obstructive CAD on CCTA and who received a S-CMR. The presence of inducible ischemia and LGE were independent predictors of the primary outcome [17].

Moreover, S-CMR was shown to be an efficient diagnostic tool for significant in-stent restenosis: in two different studies, S-CMR showed a high diagnostic accuracy above 90% in detecting in-stent restenosis on a per-vessel level [18,19].

All of these data contributed to establishing S-CMR as a valuable diagnostic and prognostic tool to the point that it is currently recommended by international guidelines in the assessment of patients with known or suspected CAD [3,20].

### 2.2. How S-CMR

The S-CMR protocol, which should be performed according to the latest update of the S-CMR guidelines [21], consists of stress and rest phases, with final LGE sequences. In order to assess myocardial ischemia, S-CMR can be performed either using vasodilator drugs (dipyridamole, adenosine or regadenoson) or inotropic agents (dobutamine). 

With the administration of a short half-life vasodilator stress agent, such as adenosine, perfusion defects can be outlined, while with long half-life vasodilator stress agent administration, such as dipyridamole and regadenoson, both perfusion defects and regional wall motion abnormalities (WMA) can be revealed. The depicted perfusion defect is a result of a “steal phenomenon” and loss of autoregulation mechanism induced by adminisering these drugs. Vasodilator agents should not be used in case of second-degree (type 2) or complete atrioventricular block, sinus bradycardia (<40 beats/min), low systolic blood pressure (<90 mmHg) or severe systemic arterial hypertension (>220/120 mmHg), active bronchoconstrictive or bronchospastic disease with regular use of inhalers, and known hypersensitivity to adenosine, dipyridamole, or regadenoson. An extensive list of contraindications is listed in Table 2. However, regadenoson is known to be better tolerated, having the advantage of being administered without the use of an infusion pump and in weight-independent dosing.

Moreover, S-CMR is able to show WMA mediated by an increase in heart rate after inotropic agent administration, such as dobutamine [22]. Dobutamine stress CMR should not be performed in patients with serious hypertension (>220/120 mmHg); unstable angina pectoris; uncontrolled heart failure; severe aortic stenosis; obstructive hypertrophic cardiomyopathy and complex cardiac arrhythmias, including uncontrolled atrial fibrillation; and in patients with myocarditis, endocarditis, or pericarditis [23]. Preliminary experiences also show promising results in ischemia assessment during physical exercise through use of a magnetic resonance compatible ergometer bicycle [24,25]. In clinical practice, vasodilator stress perfusion testing is the most common choice [21].

**Figure 1:**
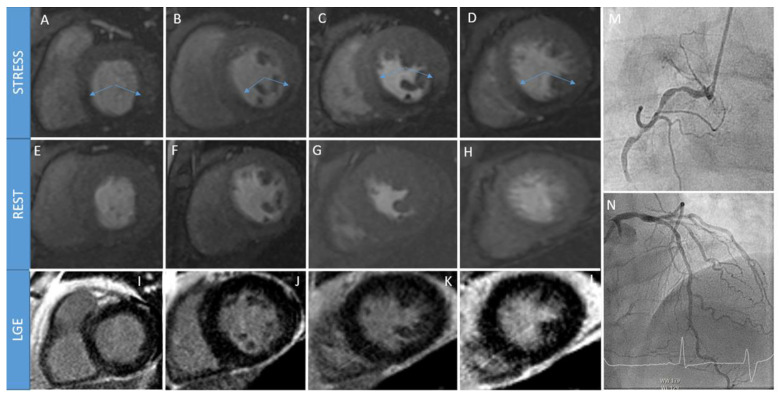
A 68-year-old patient with stable chest pain undergoes stress perfusion cardiac magnetic resonance (S-CMR). Stress slices (panels (**A**–**D**)) highlight presence of perfusion defects (see arrows) in inferior, infero-septal, and infero-lateral walls that are not present during rest phase (panels (**E**–**H**)). Absence of scars is also noted in late gadolinium enhancement (LGE) sequences (panels (**I**–**L**)). Invasive coronary angiography (ICA) confirms presence of a chronic total occlusion of right coronary artery (panel (**M**)), with some collaterals coming from left anterior descending artery (panel (**N**)).

Once scout images are performed, hyperemia is induced and a gadolinium-based contrast agent is administered, which usually occurs during the last minute of adenosine or dipyridamole infusion and 45–60 s after regadenoson injection [21]. T1-weighted CMR images are acquired during every heartbeat to evaluate the transit of the gadolinium throughout heart chambers and the perfusion of the myocardium. Moreover, the acquired images need to include the basal, mid, and apical segments of the left ventricle on the short axis in order to evaluate all of the 16 myocardial segments as standardized by the American Heart Association (AHA) [7]. The contrast agent enters normally perfused myocardium faster and at higher concentrations, resulting in a more enhanced T1-signal in comparison to abnormally perfused myocardial segments. The finding of hypointensity along the subendocardium in a coronary distribution is specific to a perfusion defect and is typically highlighted 2–3 heartbeats after the left ventricle cavity reaches its maximal contrast-enhancement [26]. After approximately 15 min, the resting perfusion images with the same described technique and slice location are acquired. After waiting another 5 min to allow for washout of the gadolinium from the myocardium, LGE images are acquired to assess viability. The described protocol lasts around 30 min, and an example of a positive S-CMR is illustrated in Figure 1.

In the case of inotropic stimulation, cine-CMR images are acquired in three distinct long-axis planes and three short-axis planes for every incremental dosage of dobutamine for the qualitative assessment of WMA.

Analysis of S-CMR perfusion images starts from a dynamic series of images that follows contrast perfusion into the myocardium [21]. As mentioned previously, a perfusion abnormality will be displayed as a hypointensity in the subendocardial layer with a coronary distribution, with the defect being most evident 2–3 heartbeats after the left ventricular cavity is maximally enhanced with contrast and persisting for a few more seconds during the contrast wash-out. Perfusion images must be compared to LGE images to distinguish between myocardial ischemia and myocardial infarction in the segments affected by a perfusion defect. Moreover, the differentiation from imaging artifacts is fundamental. Among them, dark rim artifacts are the most potentially misleading and are typically recognized as thin subendocardial perfusion defects that are present during both resting and stress images, without concomitant LGE. Moreover, a dark rim tends to be circular, while a real perfusion defect is typically focal segmental and visible for several image frames. 

Even though, in common clinical practice, the analysis of S-CMR images is performed visually, semiquantitative and quantitative evaluation of myocardial ischemia may be helpful in more challenging situations, such as left main and multivessel disease or microvascular dysfunction [27,28]. Quantification of the myocardial signal during first-pass perfusion reflects the absolute value of myocardial blood flow in each pixel of the image and may be obtained using several approaches, such as the uptake model, one-compartment model, Fermi model, and deconvolution method. These methods can be simply described as follows:

- uptake model: this model measures myocardial blood flow by analyzing the uptake of a contrast agent in the myocardium. It involves calculating the rate of contrast agent accumulation in the tissue.
- one-compartment model: this model assumes that the myocardium can be represented as a single compartment. It estimates myocardial blood flow by analyzing the contrast agent concentration over time within this compartment.
- Fermi model: the Fermi model is a mathematical model used to analyze the contrast agent concentration data in the myocardium. It incorporates both the arterial input function and the tissue residue function to estimate myocardial blood flow.
- deconvolution method: this method involves deconvolving the contrast agent concentration curve in the myocardium with the arterial input function. It aims to extract the impulse response function, which represents the transfer of the contrast agent through the tissue, thus allowing for the estimation of myocardial blood flow.

Recent studies suggest the improved diagnostic and prognostic utility of pixel-wise quantification of myocardial blood flow in comparison to solely using visual evaluation. Ischemic burden in a certain coronary territory detected using a quantitative method may be greater than what is appreciated with solely visual analysis [29]. Moreover, Sammut et al. found that quantitative analysis provided incremental prognostic value to visual assessment and established risk factors, potentially representing an important step forward in the translation of quantitative CMR perfusion analysis into the clinical setting [30]. 

In the setting of ischemia in non-obstructive coronary disease, the CMR-derived myocardial perfusion reserve index (MPRI) also serves as a solid semiquantitative value representing the vasodilating capacity of small vessels, being defined as the ratio of stress to rest upslope normalized to the upslope of the left ventricular (LV) blood pool [31]. Liu et al. also defined, for MPRI, a cutoff threshold of 1.4 for the diagnosis of microvascular angina, with an accuracy of 92% [32]. More recently, a study showed that MPRI measured with S-CMR is an independent predictor of major adverse cardiovascular events (MACE). Patients with MPRI < 1.47 had a three-fold increased risk of MACE compared with those with MPRI > 1.47 [33].

### 2.3. When S-CMR

The selection of the preferred diagnostic tool to investigate CAD is guided by several factors, such as the pre-test probability of CAD, the patient’s comorbidities (renal function, presence of cardiac devices and arrhythmias, etc.) and the availability, expertise, and preference of each center. However, it is expected that for each test, there is a range of pre-test probability of significant CAD within which its performance is maximized. In their meta-analysis, Knuuti et al. defined the ranges of pre-test probability of CAD where the different techniques were able to rule in or rule out significant CAD by driving the post-test probabilities above 85% and below 15%, respectively. This method was performed for both anatomically and functionally significant CAD, taking as a reference standard ICA and invasive FFR, respectively [4]. From these data, it appears that S-CMR performs most effectively in the rule-in of patients with intermediate-to-high clinical likelihood of CAD, both for anatomically and functionally significant CAD. Moreover, it is known that revascularization decision-making requires the evaluation of ischemia in most patients, with functional imaging, including S-CMR, demonstrating an ability to reduce referrals for ICA compared with a strategy based on anatomical imaging [12]. Nonetheless, the Dan-NICAD trial showed low sensitivity (28–41%) of S-CMR in detecting hemodynamically significant CAD defined via invasive FFR in patients with suspected obstructive CAD based on CCTA [34]. These results are in contrast with the previous literature and might occur due to patient selection, as 61% of patients had angiographically intermediate lesions and the mean FFR of the study was close to the cut-off for hemodynamic significance (0.83). 

Considering the data in our possession, S-CMR may be preferred in patients with a higher clinical likelihood of CAD or who were previously diagnosed with CAD. Current ESC guidelines on CCS published in 2019 received these data, making an important step forward in comparison to the previous version [3,35]. Similarly, 2018 ESC guidelines on myocardial revascularization and 2021 ESC guidelines on heart failure (HF) recommend the use of stress CMR for the evaluation of ischemic segments and myocardial viability in patients with heart failure and CAD to decide whether or not they should undergo myocardial revascularization (class IIb, level of evidence B) [36,37]. Eventually, the 2020 ESC guidelines on acute coronary syndrome without ST-elevation recommend using stress-CMR for patients with normal ECG and no elevation of high-sensitive troponin, but are still suspected as having acute coronary syndrome, before performing invasive tests (class I, level of evidence A) [38].

## 3. Myocardial Computed Tomography Perfusion

### 3.1. Why CTP

While the sensitivity of CCTA is excellent, the specificity of CCTA is unsatisfactory, with serious risk of an overestimation of the severity of CAD, especially in the intermediate range (40–80%) stenosis. Indeed, in their meta-analysis, Danad et al. found the sensitivity of CCTA to be 90%, while the specificity was only 39% in evaluating hemodynamically significant lesions, as defined by FFR measurement [14]. In contrast, other tests, such as stress echocardiography, SPECT, S-CMR, and PET, evaluate the presence of ischemia but fail in providing detailed information about anatomy, including atheroma burden and its composition. The EVINCI data demonstrated that a combination of anatomical and functional non-invasive tests avoided unnecessary ICA. Referring patients to an invasive procedure with the combination of positive CCTA and stress test also translated into a reduction in major adverse cardiovascular events (MACE) and cost-effectiveness [39,40]. 

In the last few years, developments in the CCT technology answered the request of combining anatomical and functional evaluation. The advancement of myocardial CTP lies in the possibility of a “one stop shop” strategy, adding incremental predictive value to the sole CCTA-based coronary stenosis evaluation. CTP detects the presence of inducible ischemia following the administration of a hyperemic stimulus, and, if present, reduced perfusion is depicted as an area of hypodensity. In clinical practice, a pharmacological stressor is administered to induce ischemia, i.e., either adenosine or regadenoson, and a CCTA as a rest phase can eventually precede or follow, according to the patient’s risk profile and physician’s preference.

The regadenoson crossover study demonstrated regadenoson CTP to be non-inferior to SPECT in the detection of inducible ischemia. The combination of regadenoson CCTP and CCTA raised the diagnostic accuracy from 69 to 85%, with sensitivity and specificity being 90% and 84%, respectively [41]. The prospective multicenter CORE320 trial demonstrated that, both in patients with and without a history of CAD, the combination of adenosine CTP and CCTA increased the diagnostic accuracy and specificity in detecting hemodynamically significant coronary stenosis, which is defined as ≥50% stenosis via ICA and was responsible for a perfusion defect via SPECT. Moreover, CTP imaging was more accurate in the diagnosis of anatomic CAD (stenosis ≥ 50% at ICA using quantitative methods) than SPECT due to a higher sensitivity for left main and multivessel disease [42,43]. Starting from the results of the CORE320 trial, a sub-study showed that the diagnostic performance of CTP was similar to that of S-CMR [44]. 

Importantly, CTP even showed its diagnostic efficacy in groups of patients who present the greatest challenge to CCTA, such as patients with a high pre-test probability of CAD, severely calcified lesions, or coronary stents. In patients with coronary stents, CTP significantly improved the diagnostic rate and accuracy of CCTA alone when compared with both invasive coronary angiography and invasive FFR as the gold standard [45,46]. Moreover, a meta-analysis showed that CTP was better than SPECT and stress echocardiography and comparable to S-CMR and PET for the detection of myocardial ischemia, even at the patient level, using FFR as a reference standard [47]. Celeng et al. demonstrated CTP to have high sensitivity and specificity for the detection of hemodynamically significant CAD [48]. Similarly, Pontone et al. reported a sensitivity and specificity of CTP compared to invasive FFR of 73–80% and 86–90%, respectively. However, when CTP is acquired after a CCTA, the sensitivity and specificity rise to 74–83% and 88–93%, respectively [15]. 

Regarding the prognostic value of CTP, the combination of CCTA and CTP imaging resulted in a similar prediction of MACE at 2 years (defined as revascularization, myocardial infarction, hospitalization for chest pain or congestive heart failure, arrhythmia, or cardiac death), late MACE, and event-free survival to that obtained with the use of both SPECT and ICA [49]. Van Assen et al. also demonstrated that stress CTP has a higher prognostic value than coronary CTA or Fractional Flow Reserve Derived from CT (FFR_CT_) for the prediction of major adverse cardiac events [50].

### 3.2. How CTP

In the last few decades, CCT became the protagonist of dramatic evolutions in terms of technology, radiation dose and time of acquisition reduction, and overall performances. Currently, a 64-slice CT scanner is considered the minimum technology required for CTP imaging. 

**Figure 2:**
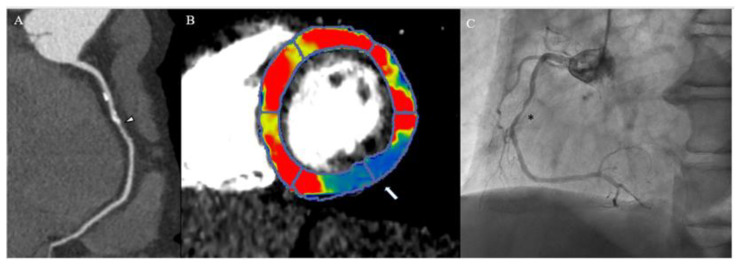
A 65-year-old male patient with a history of chest pain underwent a coronary computed tomography angiography, showing a stenosis of mid-right coronary artery (arrowhead, panel (**A**)). Computed tomography myocardial perfusion showed a perfusion defect of inferolateral and inferior walls marked as a blue region in comparison to normal perfused myocardium in red and yellow (arrow, panel (**B**)). Invasive coronary angiography confirmed a severe stenosis of right coronary artery (asterisk, panel (**C**)).

Similarly to other functional stress tests and according to the indications given by the Society of Cardiovascular Computer Tomography, CTP comprehends a series of rest and stress imaging acquisitions [51]. However, in this case, iodinate contrast agent is used and injected at a rate of at least 5 mL/s to achieve the enhancement in the first-pass arterial phase. Moreover, differently from S-CMR and SPECT, the rest scan in CTP is of key importance, as it represents the moment of evaluation of both coronary stenosis and myocardial perfusion, adding anatomical information to an otherwise functional evaluation, as shown in Figure 2. 

The stress scan involves the intravenous administration of vasodilator drugs (e.g., adenosine, dipyridamole or regadenoson) to assess inducible ischemia. A following late-enhancement scan is optional and usually performed to evaluate viability in selected cases, taking place 5–10 min after the contrast injection. The decision about whether to opt for a stress/rest or rest/stress protocol is usually patient-tailored [51,52]. A rest-first protocol should be preferred in patients at low-to-intermediate pre-test probability of obstructive CAD, since rest images not suggestive of obstructive CAD permit us to avoid following stress, which reduces the radiation and contrast medium exposure. In the case of a patient with a known coronary anatomy or high pre-test probability of CAD, a stress-first protocol is preferable, as it avoids reduction in sensibility due to contrast impregnation of the myocardium during the rest phase. Irrespective of the chosen protocol, an interval of 10–15 min between the scans is performed to ensure a sufficient contrast wash-out.

Currently, two different techniques are available for CTP: static and dynamic CTP acquisitions. 

Static CTP imaging acquires one single phase dataset during the first pass of the contrast agent in the myocardium, depicting the myocardial perfusion at one precise time point. This approach means that the timing of the scan acquisition is fundamental and requires careful assessment, with the objective being to acquire the images at the highest contrast-to-noise ratio difference between the normal and hypoperfused myocardium [53]. As per CCTA, CTP can be conducted via a retrospective ECG-gating or a prospective ECG-triggering method, without a definitive preference between the two options, even though the retrospective method allows for lower radiation dosing and smaller CT-detectors. The evaluation of the contrast enhancement is mainly performed qualitatively, with perfusion defects appearing hypodense compared to the surrounding normal myocardium, and usually being distributed at the subendocardium or transmural in nature. Similar to nuclear imaging, the comparison of stress and rest images allows for the distinction between inducible ischemia, if the hypo-attenuation is reversible at rest, and scar due to prior myocardial infarct, if the perfusion defect is fixed. Eventually, if the hypoperfusion at stress is still appreciated at rest, albeit reduced in extension, peri-infarct-ischemia can be diagnosed. Static CTP also allows a semi-quantitative evaluation, allowing for the measurement of the transmyocardial perfusion ratio, which is defined as the ratio of endocardial-to-epicardial attenuation.

Dynamic CTP consists of the acquisition of multiple imaging datasets at different time points during a 20–30-s-long inspiratory breath-hold. Dynamic CTP can be obtained via a prospective ECG-gated dynamic acquisition in the case of a CT scanner with a large coverage on the z-axis (256- or 320-detector-row) or, if detectors are narrower, an ECG-triggered axial shuttle mode technique with a second- or third-generation dual source CT scanner. The acquired images are used to create time-attenuation curves (TACs) for each voxel of the myocardium and the arterial input function (AIF), which are derived from the left ventricle or the thoracic aorta. Using a dedicated parametric deconvolution based on a two-compartment model of intra- and extra-vascular space to fit the TACs, dynamic CTP imaging enables absolute quantification of myocardial perfusion, such as myocardial blood flow (MBF, mL/100 mL/min), MBF ratio, and myocardial blood volume (mL/100 mL). Finally, through comparing MBF during stress and rest phases, an assessment of absolute coronary flow reserve may be obtained. The most appealing aspect of dynamic stress CTP is its quantitative approach, which makes reporting less operator-dependent and more reproducible compared to static stress CTP, especially in challenging settings, such as multivessel obstructive coronary disease or microcirculation dysfunction. 

Compared to static CTP, the radiation exposure of a dynamic approach is higher, ranging between 8 and 9 mSv for the “shuttle-mode” technique and being 5 mSv for “whole-heart coverage” scanners. Notably, according to a recent pooled analysis on a per-patient basis, dual-energy and dynamic quantitative CTP tends to have a slightly higher sensitivity than static CTP imaging [54]. This finding may be related to higher detection of subtle perfusion defects. Table 3 summarizes the differences between static and dynamic CTP. However, experience from the PERFECTION (Stress Computed Tomography Perfusion Versus Fractional Flow Reserve CT Derived in Suspected Coronary Artery Disease) study revealed that both static and dynamic CTP increased diagnostic accuracy, in combination with CCTA, in detecting functionally relevant coronary stenoses [55,56]. 

Moreover, when compared to S-CMR, dynamic CTP shows similar diagnostic accuracy. For instance, de Knegt et al. [57] demonstrated that coronary computed tomography angiography (CCTA), visual stress cardiac magnetic resonance (S-CMR), and CCTA and relative computed tomography myocardial blood flow (CT-MBF) had better sensitivity compared to CCTA and visual computed tomography perfusion (CTP), with similar sensitivities for CCTA and visual s-CMR perfusion and CCTA and CT-MBF. Regarding specificity, there were no differences between these three techniques [57]. Similarly, in a meta-analysis by Takx et al., the diagnostic accuracy of dynamic CTP (AUC 0.93) was comparable with S-CMR (AUC 0.94) on a per-vessel level [47].

### 3.3. When CTP

An important limitation to myocardial CTP implementation is the heterogeneity within the literature of pharmacologic stress agents, imaging sequences, scanner types, acquisition protocols, post-processing, and interpretation of CTP results. Clinical adoption of myocardial CTP is further hindered by the absence of an expert consensus regarding when and how CTP should be performed. Since CCTA alone has a very high negative predictive value to exclude myocardial ischemia in the presence of no CAD or a non-obstructive stenosis (≤50% severity), selection of a myocardial CTP protocol should generally be reserved for a situation in which the presence of ischemia after performing CCTA is doubtful or known to be difficult to evaluate, such as coronary artery stenoses of unknown hemodynamic significance, severe coronary calcification, or coronary stents [58,59]. The Society of Cardiovascular Computed Tomography recommends adding CTP to standard CCTA if it is known that the presence and severity of ischemia would impact patient management [51]. This approach refers to cases with a high pre-test probability for obstructive CAD, including those patients with prior coronary intervention or significant calcification, as well as cases when there is a stenosis of indeterminate functional significance. Finally, CTP requires higher radiation and contrast doses and longer scan times. Hence, the use of CTP is limited in patients of young age or with kidney disease. Moreover, it shares similar contraindications to those listed in Table 1 for S-CMR, as the same vasodilator agents are used in both techniques. 

## 4. Future Directions

Multiple studies enhanced the increasingly important role of additional mapping sequences in CMR protocols, while, potentially, the application of mapping sequences may help detect myocardial ischemia [60]. Stress native T1-mapping could potentially target changes in native myocardial T1 values under vasodilation stress (“T1 reactivity”), reflecting alterations in MBF due to inducible ischemia. Moreover, myocardial strain imaging is widely known to permit the detection and quantification of changes in LV function before the occurrence of LVEF reduction [61]. More recently, it was also shown that, in patients with a suspected CAD, a reduction in global longitudinal strain at peak myocardial hyperemic stress can be associated with perfusion defect [62]. Palmisano et al. evaluated both adenosine S-CMR mapping and strain data in a small cohort of patients who received a coronary sinus Reduce device. After implantation, an improvement in myocardial perfusion and both longitudinal and circumferential strain was observed, even without significant changes in strain rate and native T1 and extracellular volume (ECV) [63]. Advantages of T1 mapping and feature tracking analysis in S-CMR include the existence of a non-invasive and quantitative measure of myocardial-inducible ischemia without the use of contrast agents. It is reasonable to believe that these emerging imaging techniques will be considered complementary when performing a single CMR. Artificial intelligence and deep-learning algorithms could be crucial to fully analyzing the data that can be obtained from a single CMR examination.

Apart from the newest and most promising technologies, S-CMR still suffers from long scanning times and some clinical limitations, such as claustrophobia, metal devices, patients with clinical instability or arrhythmias, and the use of contrast agents. Moreover, evaluation of the different pharmacological agents used to induce perfusion defects or wall motion abnormalities is still lacking, and a comparison between them is needed. A scientific effort must be made to fill these gaps and overcome the technical and clinical issues related to the method.

As per CT, at present, a great variability in tools is already offered to cardiologists and radiologists for the visualization of myocardial ischemia. However, the contrast agent use and the high radiation dose when the most accurate techniques are used, such as dynamic CTP, remains an issue. Scientific advances are needed to aim for a reduction in the dose with low tube voltage imaging or the diffusion of dual-energy technique.

Over the past five years, photon-counting computed tomography (PCCT) emerged as a new modality with improved spatial resolution and soft-tissue contrast and reduced noise, blooming, and beam-hardening artifacts [64]. This outcome is the result of using new energy-resolving detectors, known as photon-counting detectors (PCDs), that register separately the energy of each photon, thus allowing a better measurement of the transmitted spectrum. Moreover, PCDs are made of a smaller pixel size than the detectors of conventional CT, leading to a higher resolution [65]. A recent prospective study was the first to compare PCCT to conventional CCTA in humans, comparing the imaging quality of the two techniques. Fourteen participants with CAD underwent retrospective CCTA with both systems. Scores of overall quality and diagnostic confidence were higher with PCCT imaging and proportions of improvement with PCCT images for quality of calcification, stent, and non-calcified plaque were 100%, 92% (95% CI: 71, 98), and 45% (95% CI: 28, 63), respectively [66]. Trials that include more patients and investigate the diagnostic accuracy of stenosis measurement are needed to further validate this promising technique.

## 5. Conclusions

Functional imaging with either S-CMR or CTP gained ever-more consideration in the last decade, with S-CMR also being included in the diagnostic path of patients with suspected or known CAD in the 2019 ESC guidelines for CCS management [3]. For a patient-tailored approach, the choice of either technique should be based on single clinical needs, cost-effectiveness, local expertise, and availability. In younger patients, S-CMR may be preferred to CTP, with the former technique being able to detect inducible myocardial ischemia and infarction, motion wall abnormalities and LGE with high specificity and sensibility without the need for ionization radiation. Moreover, the excellent prognostic role of S-CMR should not be underestimated, as it is the most promising tool to date for predicting acute cardiovascular events, although improved risk stratification is needed [2].

Nowadays, CTP is less considered and used in clinical practice; however, the possibility of acquiring functional imaging immediately after anatomical evaluation with CCTA is attractive and could lead to a possible revolution in functional imaging. Moreover, it could be a better choice in patients who are not fit for S-CMR, as in cases where claustrophobia or the presence of cardiac implantable devices are complicating factors.

In conclusion, S-CMR and CTP are both excellent diagnostic and prognostic tools that need to be further investigated to enable even more accurate and personalized management of patients with CAD.

## Figures and Tables

**Table 1:** Main characteristics and limitations of main imaging methods used for ischemia assessment. Definitions: stress transthoracic echocardiography (S-TTE); single-positron emission computed tomography (SPECT); stress cardiac magnetic resonance (S-CMR); positron emission tomography (PET); computed tomography perfusion (CTP); not applicable (NA).

	S-TTE	SPECT	S-CMR	PET	CTP
Radiation	NA	++	NA	++	+++
Nephrotoxicity	NA	NA	+	NA	++
Specificity	++	++	+++	+++	+++
Sensitivity	+	++	++	+++	+++
Diagnostic accuracy	+	++	+++	+++	+++
Temporal resolution	+++	++	++	++	+
Spatial resolution	++	++	+++	+++	+++
Availability	+++	+++	++	+	+
Cost	+	++	+++	+++	+++

+: low; ++: moderate; +++: high.

**Table 2:** List of contraindications (CI) to use of vasodilator agents.

Absolute CI	Relative CI
High-risk acute coronary syndrome	Left main stenosis > 50%
Decompensated heart failure	Outflow tract obstruction
Blood pressure > 200/110	Electrolytes alterations
Hypotension < 90	Significant tachy-/bradyarrhitimias
Bronchospastic lung disease	Recent stroke or seizure
Severe aortic stenosis	Heart rate > 100 bpm
Uncontrolled arrhythmia	Moderate renal insufficiency
Bronchospastic lung disease	Morbid obesity
Acute pulmonary embolism	
Active cerebrovascular accident	
Acute myocarditis or pericarditis	
Acute aortic dissection	
Severe renal insufficiency	
High degree atrioventricular block	
Caffeine, chocolate, or theophylline in the last 12 h	

**Table 3:** Focus on cardiac computed tomography perfusion (CTP). Advantages and disadvantages of static and dynamic CTP. Coronary computed tomography angiography (CCTA).

CTP	Advantages	Disadvantages
Static	Rapid	No quantitative data
	Lower radiation dose More data on accuracy and outcomes	More artifacts
	CCTA and CTP in a single data set	Time of acquisition critical
Dynamic	Quantitative data possible	Compliance (long breath-hold)
	Fewer artifacts	Longer analysis
	Timing of acquisition less critical	Higher radiation dose
		Increased image noise

## Data Availability

No new data was created or analyzed in this study. Data sharing rules are not applicable to this article.
